# Weeds in the Alfalfa Field Decrease Rhizosphere Microbial Diversity and Association Networks in the North China Plain

**DOI:** 10.3389/fmicb.2022.840774

**Published:** 2022-03-17

**Authors:** Chao Yang, Wei Tang, Junqi Sun, Haipeng Guo, Shusheng Sun, Fuhong Miao, Guofeng Yang, Yiran Zhao, Zengyu Wang, Juan Sun

**Affiliations:** ^1^College of Grassland Science, Grassland Agri-Husbandry Research Center, Qingdao Agricultural University, Qingdao, China; ^2^Key Laboratory of National Forestry and Grassland Administration on Grassland Resources and Ecology in the Yellow River Delta, Qingdao, China

**Keywords:** alfalfa field, weed management, rhizosphere, microbial diversity, ecological network

## Abstract

The competition between weeds and crops for soil nutrients is affected by soil microorganisms, which drive diverse ecological processes and are critical in maintaining the stability of agroecosystems. However, the effects of plant species identity, particularly between forage and weed, on soil microbial diversity, composition, and association are not well understood. Here, we investigate the soil physicochemical properties and bacterial/fungal communities in an agroecosystem with native alfalfa [*Medicago stativa* (Ms)] and five common weed species (*Digitaria sanguinalis*, *Echinochloa crusgalli*, *Acalypha australis*, *Portulaca oleracea*, and *Chenopodium album*) in the North China Plain. The five weeds had a lower plant carbon content than Ms. while the opposite was true for plant nitrogen and phosphorus concentrations. The Shannon diversity of bacterial and fungal communities of the five weeds were significantly lower than in Ms. Soil pH and PO_4_^3−^-P were identified as the most important factors in shaping the relative abundances of bacteria (Sphingomonadaceae) and fungi (Pleosporaceae), respectively. Importantly, the weeds greatly inhibited the growth of pathogenic fungi (Nectriaceae and Pleosporaceae). Bacterial co-occurrence networks depended on specific species, indicating that Ms. harbored co-occurrence networks that were more complex than those in the bacterial communities of other weed groups. Our study examines how soil nutrients and the soil microbial community structure of five weed species changed in an Ms. field. This analysis of the microbial ecological network enhances our understanding of the influence of weeds on the soil microbiome in agroecosystems.

## Introduction

Alfalfa [*Medicago sativa* L. (Ms)] is a high-quality legume forage crop with high productivity, palatability, and quality. The alfalfa industry has developed quickly in China since 2008 in response to extensive protein feed shortages nationwide (Mao et al., 2018). However, weed invasion is a serious problem in alfalfa cultivation, especially in poor-growing alfalfa fields. Generally, weed presence causes greater losses to crop production than the incidence of insect pests or pathogens (De Matos et al., 2019). The establishment period of perennial forages, such as alfalfa seedlings, are particularly susceptible to weed competition during the seeding year, and weeds that emerge after seeding reduce alfalfa success or cause permanent damage to its productivity throughout the life of the stand (Raoofi and Alebrahim, 2017). Previous studies report that weeds affect the stability of alfalfa stands, attributed to the competition with alfalfa for environmental resources, such as light, nutrients, and water resources (He et al., 2018; De Matos et al., 2019; Kanatas et al., 2021), which in turn decrease the yield, quality and the regulation of the soil properties and microorganisms (Hassannejad and Ghafarbi, 2014; Sardans et al., 2017).

Weed plants capable of actively increasing nutrient availability in the environment, especially through nitrogen (N) fixation or phosphorus (P) mobilization in the soil, outperform competitors in environments with low N and P availability (Sardans et al., 2017). In turn, N and P concentrations increase the phytomass of weed species and soils in nutrient-poor environments (Sardans et al., 2017). Additionally, interactions with soil microbiota may be important for competitive relations as soil microorganisms play key roles in the competitive ability of weed plants (Matos et al., 2019). The soil microbiome is influenced by above- and below-ground inputs linked to the composition of plant communities. Soil microbial communities and the capacity of plants to form associations with soil microorganisms may influence weed performance (De Matos et al., 2019). Weed communities exert species-specific effects on the structure of soil microbial communities and select microorganisms that establish positive/negative interactions, which increase their adaptability, stability and competitiveness in farmlands (Wortman et al., 2013; Matos et al., 2019). Thus, there is a greater capacity for adaptation and plant development in environments that allow for associations with soil microorganisms. The differences between the plant species composition and the available rhizodeposition alter the composition and diversity of microbial communities (Melo et al., 2014). A previous study observed the substantial effects of a weed species (e.g., *Centaurea maculosa*) on the abundance and community composition of soil microbial functional groups (Batten et al., 2006). Jordan and Huerd (2008) detected variable weed responses to soil fungi and found negative effects on the growth and competitive ability of weeds (*Echinochloa crus-galli, Setaria viridis* and *Solanum nigrum*) relative to crop species (Veiga et al., 2011). Thus, it is vital to enhance our understanding of the relationship between microbial communities and individual weed species. Co-occurrence networks among soil microbial taxa reflect the correlation between microorganisms and could be more important to ecosystem processes and functions than species diversity (Zhou et al., 2011). However, many of these observations are limited to weeds in unmanaged ecosystems, and studies on the effects of agricultural weeds on soil microbial community composition are less common, especially in alfalfa fields.

There is a close relationship between soil microorganisms and soil physicochemical properties, which determine plant health and soil fertility, as soil microbial communities largely determine the diversification of soil properties, such as pH and carbon (C) and N nutrient levels (Liu et al., 2018). Microbial distributions in the soil may correlate with nutritional contents, and different microorganisms have different requirements for soil nutrient status (Classen et al., 2016). Soil microorganisms have significant effects on plant nutrient uptake due to various microbial functions, such as the degradation of organic compounds and biogeochemical cycling of nutrients (Fierer, 2017). The ability of soil microorganisms to convert essential nutrients in the soil that are accessible for plant uptake depends on the soil environment and is characterized by various soil physicochemical properties (Xia et al., 2020). However, the extent to which the effects of these soil properties can differ in specific biomes are related to different variables, such as plant communities and cover crop. A recent study reports that plant species changes in microbial communities depend on the plant characteristics (Fadiji and Babalola, 2020). Weed species mitigate soil microbiota that colonize the rhizosphere and acquire nutrient sources unavailable to the crop, thereby creating soil conditions that favor weed species over native plants, which in turn increase the competitive ability of weeds (Massenssini et al., 2014; Matos et al., 2019). However, the relationship among arable weeds, soil properties, and soil microbial communities in alfalfa fields is not well understood.

The North China Plain (NCP) is an important grain-production area located in China where winter wheat (*Triticum aestivum* L.) and summer maize (*Zea mays* L.) are rotated with two harvests per year, which has been common practice for several decades (Wang et al., 2018). For more than 10 years, there has remained a large gap between dairy farmers’ feed and producers’ supply of alfalfa in China, especially in the NCP. Thus, introducing alfalfa into traditional grain-cropping systems provides the local livestock industry with much-needed protein-rich feedstuff and lowers environmental risks, including soil degradation and nutrient losses due to long-term intensive cereal cultivation (Huang et al., 2017; Zhang et al., 2021a). Most research on weed–crop competition has not explored changes in the soil microbial community structures of individual plant species. Moreover, the potential effects of weeds on soil microbial communities are not well understood (Corneo et al., 2013; De Matos et al., 2019). In the present study, we selected an alfalfa field and five weeds to compare the differences in soil chemical properties, microbial community structure, and the interactions among microbial community structure and chemical soil properties. This study aimed to (1) assess the impacts of specific-species on soil chemical properties and microbial composition, (2) determine the key soil properties affecting the soil microbial community structure, and (3) explore the soil underlying microbiological co-occurrence network of weeds in alfalfa fields.

## Materials and Methods

### Site Description and Sample Collection

The study was conducted using a 2-year Ms. system located in the northeast NCP (36°26′25″N, 120°4′48″E; elevation, 1 m), established in Autumn 2019. This area has a warm, temperate, continental, semihumid monsoon climate characterized by 686 mm annual average rainfall and a mean air temperature of 14°C. The soil type is brown earth. The application rates of N (urea) and P (calcium superphosphate) fertilizer in the experimental field were 50 kg N ha^−1^ and 150 kg P_2_O_5_ ha^−1^, respectively, due to the large demand of Ms. for P fertilizer. The top 0–20 cm of the soil profile before Ms. planting had a pH of 7.14, and the organic C, total N, and total P were 16.96, 0.88, and 0.45 g kg^−1^, respectively. Available N and P were 77.12 and 27.05 mg kg^−1^, respectively.

Plant and soil samples of weeds were collected from an Ms. field with an area of 1,500 m^2^ in July 2020. First, the weeds were investigated by the serpentine sampling method (six plot), and the number of the first five dominant weeds in each plot (3 m × 3 m) were counted. Crabgrass [*Digitaria sanguinalis* L. (Ds)], barnyard grass [*E. crusgalli* L. (Ec)], copperleaf [*Acalypha australis* L. (Aa)], purslane [*Portulaca oleracea* L. (Po)], and lambsquarters [*Chenopodium album* L., (Ca)] were identified with relative abundances of 33.3, 26.8, 17.8, 12, and 10.1%, respectively (Supplementary Figure S1). Five plants of each species were harvested from each plot. The plant roots were subsequently obtained with a root drill, and root-associated soil remaining on the roots were collected by shaking the plant roots. We mixed the five plants and soil samples into one repeat in each plot. Ms. samples were also collected as the control. A total of 36 samples (6 plant species × 6 repetitions) were established. Plant samples were oven-dried at 65°C for 72 h to calculate the shoot dry weight (SDW), root dry weight (RDW), and chemical properties. Soil samples were divided into two subsamples. One subsample was air-dried to analyze soil chemical properties, and the other subsample was stored in a − 80°C refrigerator for the determination of soil microbial structures.

Soil pH and electrical conductivity (EC) were measured using a glass electrode in a suspension with a soil-to-water ratio of 1:5. The plant C and N and soil total C and N were analyzed by dry combustion using an automated CHNS element analyzer (Elementar, Germany). Plant P and soil total P were determined following H_2_SO_4_–H_2_O_2_ digestion and measured using an autoanalyzer (AA3, Bran-Luebbe, Hamburg, Germany). Fresh soil samples were leached by 2 mol L^−1^ KCl solution, and the content of soil NH_4_^+^-N and NO_3_^−^-N in the filtered extracts were measured with an autoanalyzer (AA3, Bran-Luebbe, Hamburg, Germany). The soil available P (PO_4_^3−^-P) was extracted by shaking 2.5 g dry soil for 30 min at 20°C in 50 ml of a 0.5 mol L^−1^ NaHCO_3_ solution (pH, 8.5; Yang et al., 2019a).

### DNA Extraction and PCR Amplification

For the microbial extraction and determination methods, please refer to our previous study (Yang et al., 2019b). Briefly, genomic DNA was extracted from each soil sample using a FastDNA^®^ SPIN Kit for soil (MP Biomedicals, CA, United States). A total of 0.30 g soil was accurately weighed from each treatment. Soil DNA integrity was then detected by 0.8% agarose gel electrophoresis. The V3-V4 regions of the bacterial 16S rRNA gene were amplified using 338F (5'-ACTCCTACGGGAGGCAGCAG-3') and 806R (5'-GACTACHVGGGTWTC TAAT-3'). The noncoding region of the fungal internally transcribed spacer (ITS) was amplified using the ITS1 (5'-CTTGGTCATTTAGAGGAAGTAA-3') and ITS2 (5'-GCTGCGTTCTTCATCGATGC-3') primers (White et al., 1990). The PCR protocol was as follows: predenaturation at 95°C for 3 min, 27 cycles at 95°C for 30 s, annealing at 55°C for 30 s, elongation at 72°C for 45 s, and a final extension at 72°C for 10 min.

### Processing of MiSeq Sequencing Data

Purified amplicons were pooled in equimolar ratios and paired ends were sequenced on an Illumina MiSeq PE300 platform (Illumina, San Diego, CA, United States) following the standard protocols of the Majorbio Bio-Pharm Technology Co., Ltd. (Shanghai, China). Processing of the raw data and diversity indices, including Shannon and Chao1 richness, was conducted using QIIME ver. 1.3.0 (Caporaso et al., 2010). The reads were truncated to obtain an average quality score lower than 20 and a sliding window greater than 50 bp over three continuous bases; reads shorter than 300 bp were discarded. Then, UPARSE ver. 7.1 was used to cluster high-quality sequences with a 97% identity threshold into operational taxonomic units (OTUs; Caporaso et al., 2012).

### Statistical Analysis

Plant indices (SDW, RDW, PC, PN, PP, and R/S ratio), soil indices (pH, EC, TC, TN, TP, C/N ratio, NH_4_^+^-N, NO_3_^−^-N, and PO_4_^3−^-P), and bacterial and fungal α-diversity indices (Shannon and Chao 1) of the six species were assessed using a one-way analysis of variance (ANOVA) in PAST (ver. 3.25). The significance threshold was *p* < 0.05 using Tukey’s pairwise test.

A principal coordinates analysis (PCoA) based on Bray–Curtis similarity matrices was performed to identify the soil microbial β-diversity among the six species at the family level. The significance was tested by an analysis of similarities (ANOSIM) using the vegan package in R statistical software (ver. 3.6.3).

The relationship between plant and soil properties (SDW, RDW, PC, PN, PP, R/S ratio, pH, EC, TC, TN, TP, C/N ratio, NH_4_^+^-N, NO_3_^−^-N, and PO_4_^3−^-P) and the soil bacterial and fungal communities at the family level were determined by a redundancy analysis (RDA) using R statistical software (ver. 3.6.3). The significance of the effects of each variable was defined using Mantel tests (permutations = 999). The resulting significance level was tested by Mantel *r* statistic and *p* values. A Spearman correlation heat map was constructed to show the relationship between bacterial and fungal classifications and environmental variables (plants and soil). Network analysis is a powerful method for studying complex community organization, principles of community organization, and interactions among community members (Zheng et al., 2018; Li et al., 2021). Such analyses can identify keystone taxa that play critical roles in maintaining ecological functions (Ligi et al., 2014). We used the Networkx software to establish the co-occurrence networks at the family levels.

## Results

### Nutrient Characteristics of the Five Weeds and Root-Associated Soil

The relative number of the five weeds varied in the alfalfa (Ms) field (c S1). Crabgrass (Ds) had the highest relative number of the five weeds, which occupied 33.3%, followed by barnyard grass (Ec), copperleaf (Aa), purslane (Po), and lambsquarters (Ca).

**Figure 1:**
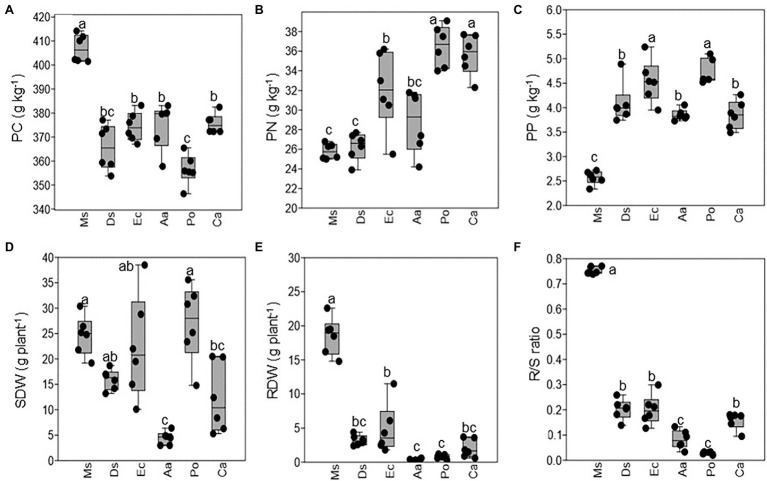
The plant nutrients and dry weights of *Medicago sativa*, *Digitaria sanguinalis*, *Echinochloa crusgalli*, *Acalypha australis*, *Portulaca oleracea*, and *Chenopodium album*. Values followed by different letters indicate significant differences (*p* < 0.05) according to Tukey’s pairwise test (PC: plant carbon; PN: plant nitrogen; PP: plant phosphorus; SDW: shoot dry weight; RDW: root dry weight; R/S ratio: root and shoot ratio).

Plant nutrients varied among Ms. and the five weeds. The plant C concentration was higher in Ms. than the five weeds, and Po had a lower plant C concentration than Aa, Ec, Ca, and Ds (Figure 1A). In contrast, both plants N and P had lower concentrations in Ms. than the five weeds (Figures 1B,C). Po and Ca had a higher plant N than the other three weeds, and Po and Ec had a higher plant N than the other weeds. The SDW, RDW, and R/S ratio varied among Ms. and the five weeds. Ms. and Po had a higher SDW than Aa or *Ca* (Figure 1D). Additionally, Ms. had the highest RDW and was higher than all five weeds. Aa and Po had a lower RDW than the other weeds (Figure 1E). Similar to RDW, R/S ratio was higher in Ms. than the weeds and lower in Aa and Po when compared with the other weeds (Figure 1F).

**Figure 2:**
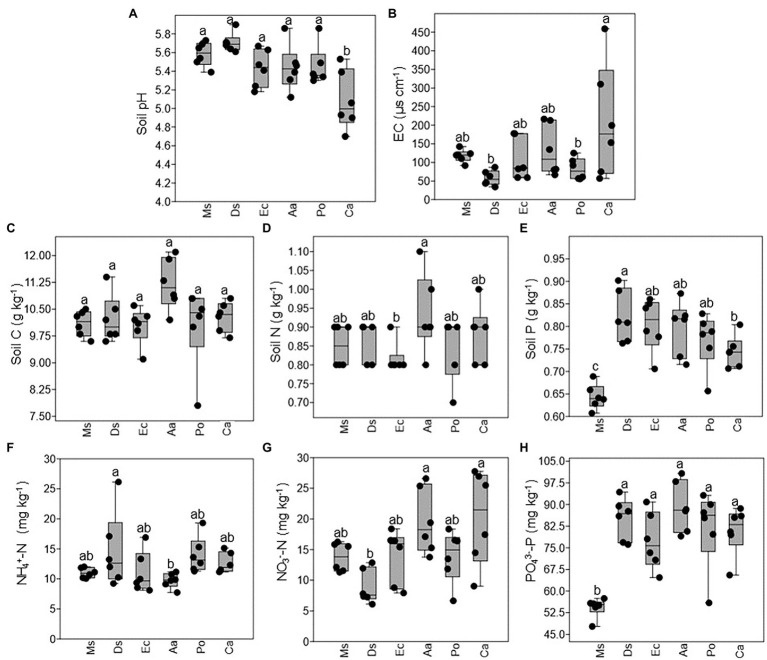
Changes in the chemical properties of rhizosphere soil collected from *M. sativa*, *D. sanguinalis*, *E. crusgalli*, *A australis*, *P*. *oleracea*, and *C. album*. Values followed by different letters indicate significant differences (*p* < 0.05) according to Tukey’s pairwise test (Soil C: soil carbon; Soil N: soil nitrogen; Soil P: soil phosphorus).

As shown in Figure 2A, the average soil pH ranged from 5.0 to 5.7 with no significant differences detected between Ms., Aa, Ec, Po, and Ds except Ca, which decreased significantly. There was no significant difference in the EC of the soils between Ms. and the five weeds (Figure 2B). Compared with Ca, the EC of the soils significantly decreased in Ds and Po. The soil nutrient contents varied with species. Soil C ranged from 10.0 to 11.1 g kg^−1^ and did not significantly differ between Ms. and the five weeds (Figure 2C). There were no significant differences in the total N, NH_4_^+^-N, and NO_3_^−^-N between Ms. and the five weeds. Compared with Aa, the soil N and NO_3_^−^-N significantly decreased in Ec and Ds, respectively while the opposite was true for NH_4_^+^-N in Ds (Figures 2D,F,G). However, the soil P and PO_4_^3−^-P in Ms. were lower when compared with all five weeds (Figures 2E,H). Compared with Ds, the soil P of Ca significantly decreased.

### The Diversity and Composition of Bacterial and Fungal Communities in Root-Associated Soil

**Figure 3:**
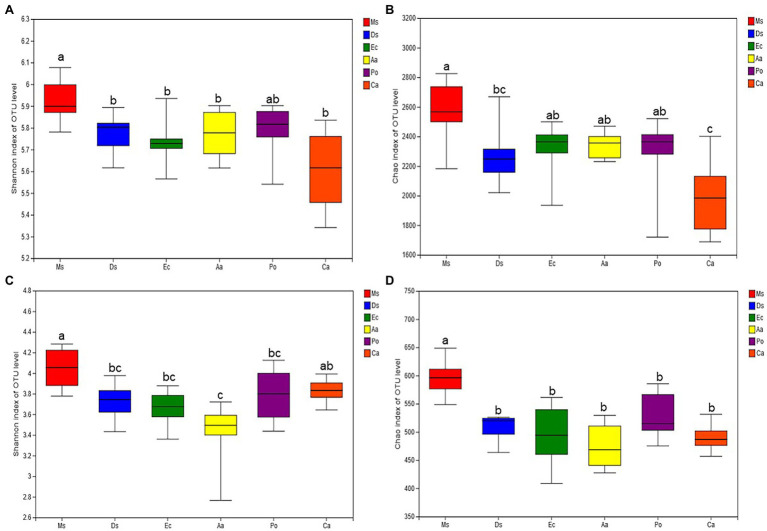
Diversity indices (Shannon and Chao 1) of soil bacterial **(A** and **B)** and fungal **(C** and **D)** communities in the six plant soil samples. All data are presented as the mean ± SD. ^*^p < 0.05 and ^**^p < 0.01 based on Tukey’s pairwise test.

Differences in the soil microbial communities were uncovered by comparing richness and diversity indices (Figure 3). For the soil bacterial communities, the Shannon diversity index was higher in Ms. than the five weeds, but no significant differences were detected among the five weeds. Similarly, Ms. had the highest Chao1 index among the six species. The Chao1 index of Ec and Aa was higher than *Ca.* For the soil fungal communities, Ms. had the highest Shannon diversity and Chao1 indices. Ca had a higher Shannon diversity index than Aa, and no significant differences were detected between the Chao1 indices of the five weeds. On average, the microbial biodiversity decreased in the five weed species when compared with Ms.

**Figure 4:**
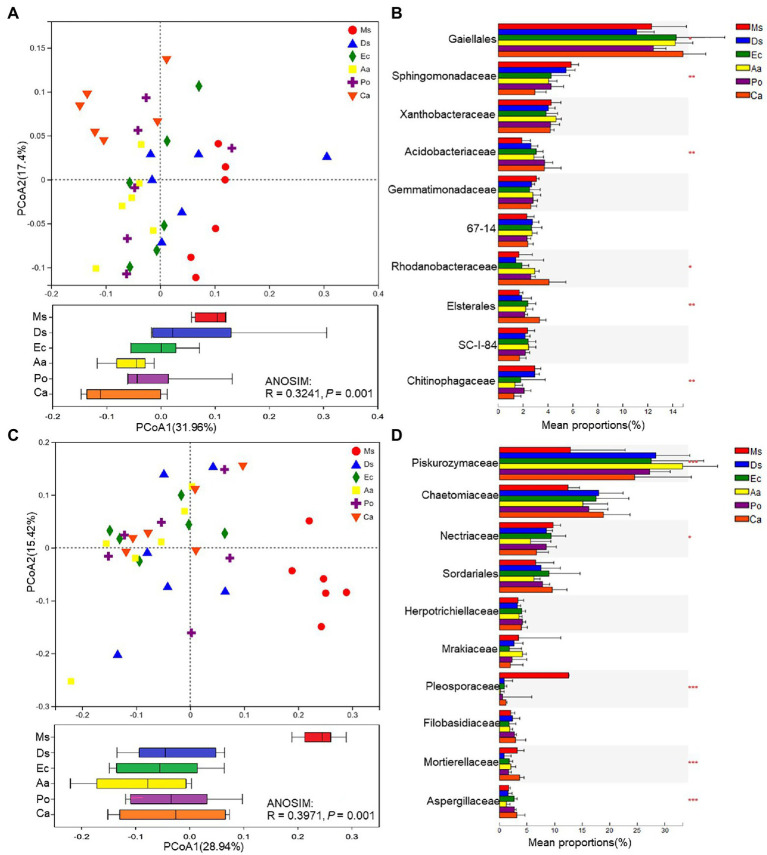
β-diversities of bacterial **(A)** and fungal **(C)** communities and relative abundances of the top bacterial **(B)** and fungal **(D)** families of the six species. A principal coordinates analysis (PCoA) based on Bray–Curtis similarity matrices was implemented to analyze the β-diversities. Ms., Ds, Ec, Aa, and Po represent *M. sativa*, *D. sanguinalis*, *E. crusgalli*, *A*. *australis*, *P*. *oleracea*, and *C. album*, respectively. ^*^p < 0.05, ^**^p < 0.01, and ^***^p < 0.001 based on Tukey’s pairwise test.

To compare soil microbial community compositions between different plant species, a PCoA based on Bray–Curtis similarity matrices was carried out. The bacterial β-diversity was significantly affected by species (*p* = 0.001; Figure 4A). The soil samples were divided into six groups; Ms. samples clustered together and were separate from Aa and Ca while there was less of a distinction between Ms. and Ds, Po, and Ec. For the bacterial communities, *Gaiellales*, *Sphingomonadaceae*, *Acidobacteriaceae*, *Rhodanobacteraceae*, *Elsterales*, and *Chitinophagaceae* were significantly (*p* < 0.05) affected by species, and *Gaiellales* was the dominant family (Figure 4B). Weeds significantly decreased the relative abundances of *Sphingomonadaceae* and *Chitinophagaceae* and increased the relative abundances of *Gaiellales*, *Acidobacteriaceae*, *Rhodanobacteraceae*, and *Elsterales* in the rhizosphere soil when compared with Ms. The fungal β-diversities varied greatly across plant species (Figure 4C). Ms. samples clustered together and were separate from the five weeds. There was less of a distinction among the weed species. The variation between fungal communities was larger than bacteria. For the fungal communities, *Piskurozymaceae*, *Nectriaceae*, *Pleosporaceae*, *Mortierellaceae*, and *Aspergillaceae* were significantly (*p* < 0.05) affected by weed species, and *Piskurozymaceae* was the dominant family (Figure 4D). Weeds significantly decreased the relative abundances of *Nectriaceae*, *Pleosporaceae*, and *Mortierellaceae* and increased the relative abundances of *Piskurozymaceae* and *Aspergillaceae* in the rhizosphere soil when compared with Ms.

### Correlations Between Soil Properties and Microbial Communities

**Figure 5:**
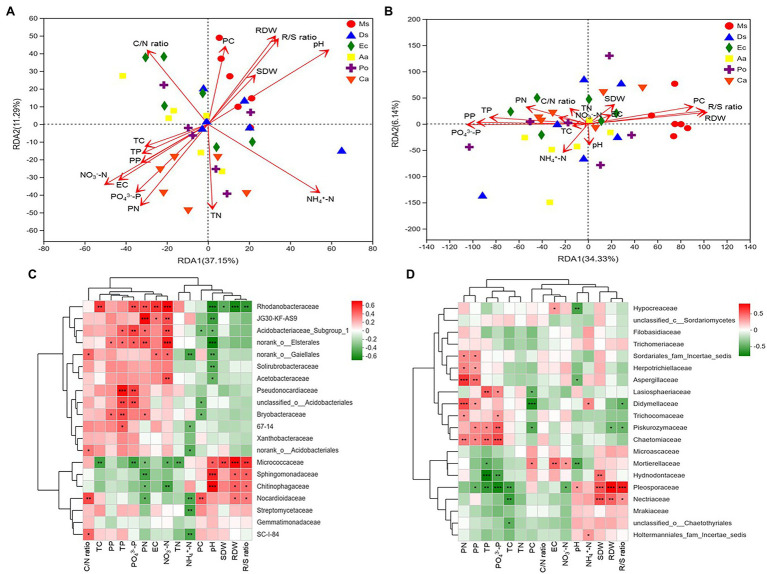
RDA plots showing the effects of plant and soil properties on the bacterial **(A)** and fungal **(B)** community structures at the family level from root-associated alfalfa and weed soil. Spearman correlation heat map showing the relationship between bacterial **(C)** and fungal **(D)** classifications at the family level and environmental variables (plants and soil). ^*^p < 0.05, ^**^p < 0.01, and ^***^p < 0.001.

As shown in the RDA biplots, a combination of variables explained 48.44 and 40.47% of the variance in the bacterial and fungal communities, respectively (Figures 5A,B). The partial Mantel test showed that PC, PN, pH, EC, NH_4_^+^-N, NO_3_^−^-N, and PO_4_^3−^-P were significantly affected by the bacterial communities, and PC, PN, PP, RDW, R/S ratio, TN, and PO_4_^3−^-P were significantly affected by the fungal communities (Table 1).

**Table 1:** The Mantel test (permutations = 999) among plant traits, soil properties, and soil microbial communities.

	Bacterial community		Fungal community	
	Value of *r*	Value of *p*	Value of *r*	Value of *p*
PC (g kg^−1^)	0.1963	0.045	0.44	0.001
PN (g kg^−1^)	0.178	0.004	0.137	0.012
PP (g kg^−1^)	0.0848	0.352	0.431	0.001
SDW (g plant^−1^)	0.0388	0.664	0.102	0.207
RDW (g plant^−1^)	0.1162	0.08	0.349	0.001
R/S ratio	0.0991	0.239	0.342	0.001
pH	0.405	0.002	−0.119	0.2
EC (μs cm^−1^)	0.28	0.01	−0.061	0.48
TC (g kg^−1^)	0.0673	0.503	0.091	0.433
TN (g kg^−1^)	0.005	0.939	−0.148	0.076
TP (g kg^−1^)	0.116	0.155	0.311	0.001
C/N ratio	0.043	0.592	0.027	0.719
NH_4_^+^-N (mg kg^−1^)	0.424	0.002	0.032	0.743
NO_3_^−^-N (mg kg^−1^)	0.3	0.004	−0.054	0.502
PO_4_^3−^-P (mg kg^−1^)	0.184	0.036	0.411	0.001

The significance level was tested by the values of *p* and *r*. PC, plant carbon; PN, plant nitrogen; PP, plant phosphorus; SDW, shoot dry weight; RDW, root dry weight; R/S ratio, root shoot ratio; EC, electrical conductivity; TC, total carbon; TN, total nitrogen; TP, total phosphorus; C/N ratio, carbon nitrogen ratio.

Pearson correlation analyses showed that PN, EC, NO_3_^−^-N, and PO_4_^3−^-P significantly positively correlated with the relative abundance of *Rhodanobacteraceae* (*p* < 0.05). The relative abundance of *Pesudonocardiaceae* significantly increased as soil TP and PO_4_^3−^-P increased (*p* < 0.05). The relative abundances of *Sphingomonadaceae* and *Chitinophagaceae* significantly increased as soil pH increased (*p* < 0.05). The relative proportions of *Pleosporaceae* and *Nectriaceae* positively correlated with SDW, RDW, and R/S ratio, and plant P, soil TP, and PO_4_^3−^-P significantly positively correlated with the relative abundances of *Piskurozymaceae*. Additionally, pH negatively correlated with the relative abundances of *Mortierellaceae* and *Aspergillaceae* (Figures 5C,D).

### Microbial Network Relationship Between Ms. and the Five Weeds in Root-Associated Soil

**Figure 6:**
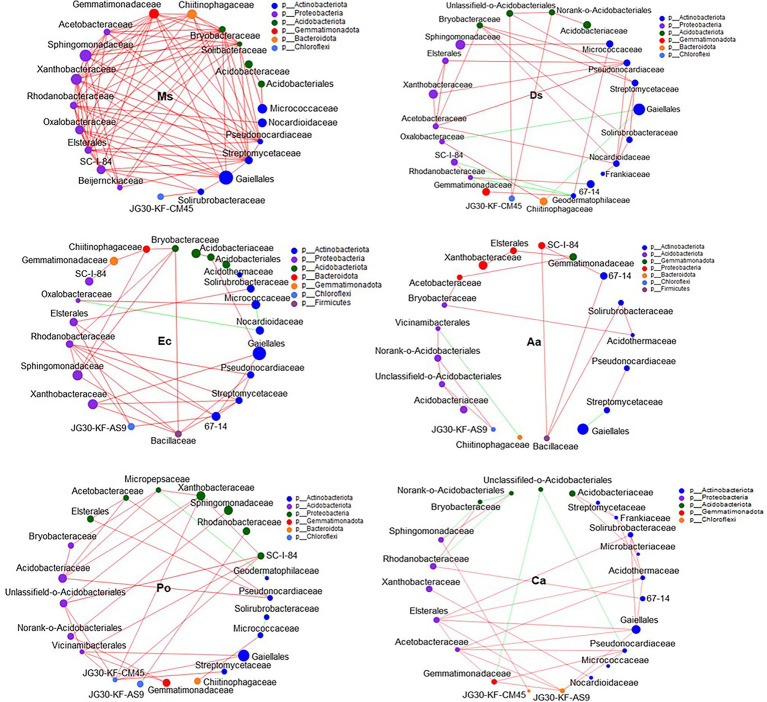
Co-occurrence patterns of soil bacteria in root-associated alfalfa and weed soil. Positive links between nodes are shown in red and negative links are shown in green (coefficient > 0.5 and *p* < 0.01).

**Figure 7:**
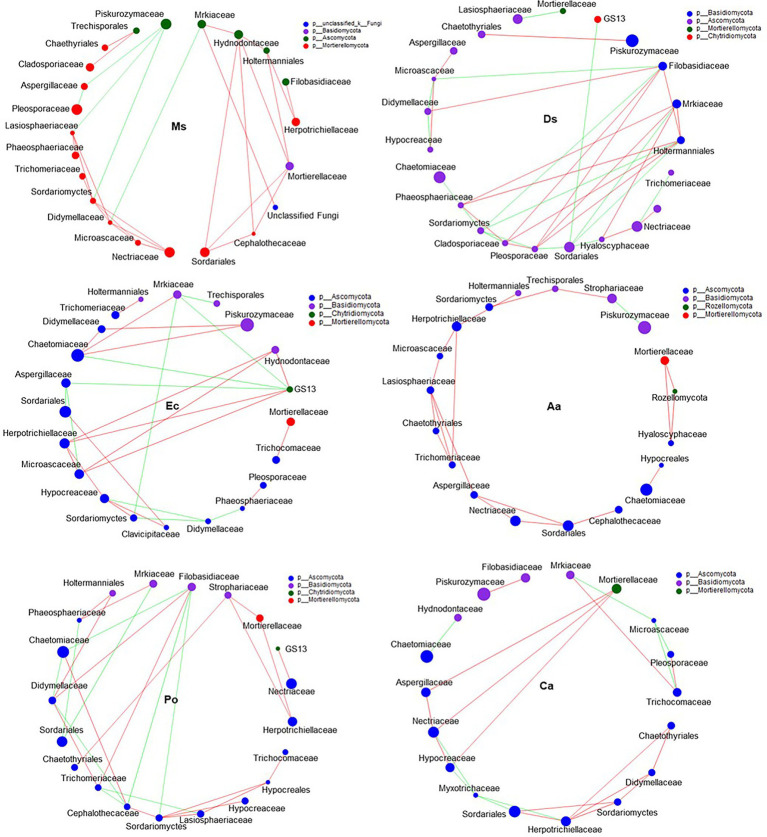
Co-occurrence patterns of soil fungi in root-associated alfalfa and weed soil. Positive links between nodes are shown in red and negative links are shown in green (coefficient > 0.5 and *p* < 0.01).

Our network results showed that the highest level of bacterial connectivity within Ms. soil had a transitivity of 0.75; the transitivity of Aa was 0.3 (Supplementary Table S1). A total of 174 positive links for bacteria were identified in Ms. soil; however, the total links decreased in weed soil, including 65, 65, 36, 58, and 68 links for Ds, Ec, Aa, Po, and Ca, respectively (Figure 6; Supplementary Figure S2). The highest level of fungal connectivity within Ca soil had a transitivity of 0.57; the transitivity of Aa was 0.35 (Supplementary Table S1). A total of 68 links for fungi were identified in Ds soil, including 52 positive and 16 negative links (Figure 7; Supplementary Figure S3).

## Discussion

### Changes in Plant Nutrients and Soil Properties

Weed species affect the soil habitat and change the plant nutrient status in native soil. The competitive capacity of a given plant species is associated with its intrinsic ability to modify nutrient accumulation to adapt to changing environmental conditions (Sun et al., 2019). Our results show that the five weeds had a lower plant C than Ms., whereas the opposite was true for plant N and plant P concentrations. A previous study indicates that Ms. maintained a high C storage capacity and accumulation due to the high efficiency of plant C assimilation and sequestration (Laubach et al., 2019). Nevertheless, weeds usually maintain high N and P for rapid leaf growth as weeds facilitate the absorption of more soil N and P to improve plant biochemical compound synthesis, respiration, and energy metabolism, thereby decreasing soil N and P availability for the growth of other plants (Sardans et al., 2017). Weeds are also associated with elevated or fluctuating nutrient resource levels by modifying soil physiochemical properties, further stimulating plant productivity (Frank et al., 2018).

The availability of plant nutrients is regulated by microorganisms through the mineralization of soil organic matter and solubilization of soil minerals (Hunter et al., 2014). Furthermore, weed establishment and persistence lead to changes in soil physicochemical properties in agricultural fields, reflecting the nutrient competitive capacity (Matos et al., 2019). In the current study, there was a distinction between the weed species and Ms. in terms of soil physicochemical properties, which were essential elements for plant growth and reproduction. The plant community also greatly affects the soil nutrient composition, directly or indirectly, by changing the ecology of the soil (Yu et al., 2021). We found that soil pH decreased in the presence of Ca, which was similar to the effects of *Spartina alterniflora* previously reported by Tong et al. (2011) as a result of changes in the levels of soil cations. Sanon et al. (2009) also found that *Amaranthus viridis* significantly increased the soil total P and soluble P when compared with native *Acacia* species. This finding is in agreement with our results; the five weeds increased the soil P when compared with Ms. Furthermore, weed roots may produce exudates containing specific allelochemicals that may enhance the solubility of insoluble soil P, thereby increasing the soil P content (Wang et al., 2015). In turn, the reduced soil P availability limits the growth of other plants (Sardans et al., 2017). The success of the weeds may be partly due to their ability to outcompete Ms. for P utilization. A previous study suggests that soil P deficiency could be a major factor in the declining root and stem biomass in Ms. (He et al., 2020). The competitive capacity of a given species facilitates the best use of limited resources and/or the ability to cope with low resource levels or reduce their availability for competitors (Ade et al., 2021). Thus, the weed species changes the plant nutrient levels and rhizosphere soil elemental composition, which in turn negatively affects Ms. growth.

### The Relationship Between Plants, Soil Parameters, and Bacterial/Fungal Community Structures

Soil microorganisms are influenced by specific species as the presence of plants controls the levels of microbial diversity and drives community assembly (Shang et al., 2021). Our results show that weeds significantly decreased bacterial and fungal α-diversity indices when compared with Ms., which were possibly elicited by inherent soil properties and the influence of plant growth on microbial communities (Cha et al., 2021). Ms. plants may enhance microbial activity in the soil due to root depositions as increased root biomass is a significant source of C for microbial biomass, which in turn regulates soil microbial communities in their immediate vicinity (Song et al., 2021).

Soil microbes play essential roles in the biogeochemical processes of nutrient cycling and serve as important links between plant species and soil interactions (Wu et al., 2021). Our results show that soil pH, NH_4_^+^-N, and NO_3_^−^-N had the significant effects on soil bacterial communities. Additionally, the fungal β-diversity significantly correlated with the root biomass and RSR of plants. C and N sources in soil ecosystems are mainly derived from the systems and plant litter, as previously shown in certain plant traits, including above−/below-ground biomass (Zhang et al., 2018). Bacteria have a broader range of physiologies than fungi and, therefore, may be less susceptible to the availabilities of C and N (Prewitt et al., 2014). In this study, shifts in the fungal composition occurred in weed species related to changes in TP and PO_4_^3−^-P. These results indicate that these soil chemical characteristics closely correlated with the bacterial and fungal communities. Soil pH is a key factor that affects bacterial community structure (Griffiths et al., 2011). Our results found positive correlations between pH and the abundance of certain bacterial genera, including *Sphingomonadaceae* and *Chitinophagaceae*, and negative correlations between pH and the abundance of *Gaiellales*, *Acidobacteriaceae*, *Rhodanobacteraceae*, and *Elsterales*. The increased abundance of *Chitinophagaceae* facilitates the degradation of polysaccharides and cellulose that promote plant growth (Bailey et al., 2013). However, *Sphingomonadaceae* is a potential pathogen found in diseased soil areas (Sanguin et al., 2009), which possibly affects the health of Ms. plants. Additionally, *Gaiellales* thrives more under weed rhizosphere conditions, which could damage the surrounding ecosystem through biological processes and increase the C and N cycles, which may be due to their greater competitive ability in stressful environments (Lin et al., 2019). *Acidobacteriaceae* were also enriched in weed soils, which is consistent with its known preference for low pH soil and specialization in the degradation of complex organic matter attributing to its lower nutrient requirements and control of disease (Tao et al., 2017). For the fungal families, weed soils were enriched with *Chaetomiaceae*, which is known to degrade recalcitrant plant material (Habtewold et al., 2020), thereby releasing plant-derived cellulose that supports the growth of bacterial and fungal communities (Eichorst and Kuske, 2012). *Nectriaceae* is a known soil-borne pathogenic family of fungi that is positively associated with certain plant traits (shoot and root biomass; Yin et al., 2021). Our results show that more harmful fungal taxa with known pathogenic traits were overrepresented in Ms. rhizosphere soil, including *Nectriaceae* and *Pleosporaceae.* Ms. roots easily suffer from *Fusarium* spp. pathogens, which pose direct negative effects on Ms. health (Wang et al., 2020). Furthermore, *Pleosporaceae* was found in greater abundance in Ms. rhizosphere soil than the five weeds. This ubiquitous and diverse fungal family contains both plant pathogens and potential post-harvest pathogens (Steenwerth et al., 2021). Thus, weeds could alter the bacterial communities in the soil to ameliorate the availability of soil nutrients and shift soil fungal communities to a good and healthy status.

### Microbial Co-occurrence Network

Generally, soil microorganisms form complex interspecies networks within ecological communities that subsequently regulate ecosystem functions (Abedini et al., 2021). A bacteria network analysis showed more nodes and links than fungi, indicating a more complex and well-connected network of the bacterial community (Banerjee et al., 2016). Furthermore, the weed microbial network analysis in this study showed lower bacterial connectivity transitivity values when compared with the Ms. network, suggesting that a sparse interaction occurs among microorganisms in weed networks, but the microbial populations of weed species are more resistant to specific species changes. Moreover, the percentage of positive links increased in the Ms. network, which in turn enriched several mutualistic microbes. Previous studies found that organic matter input can increase positive associations (De Menezes et al., 2015) and covariations are predominantly positive among rhizosphere bacteria with abundant nutrients (Shi et al., 2016). However, negative links could improve network stability as a result of competition that could stabilize co-oscillations in microbial communities (Zhou et al., 2020). In weed species, limited nutrients may increase competition and favor many trophic levels (Freilich et al., 2018). However, our results show that three weed species increased the interactions within fungal communities when compared with Ms. and significantly increased the transitivity links and percentage of positive links between microorganisms. Moreover, weeds may affect the stability of community structures that increase mutualistic relationships among fungal communities (Dawson and Schrama, 2016). As such, the dominance of fungal interactions may be explained by their ability to grow as mycelium, which confers the unique capacity to explore soil space and reallocate energy and nutrients from different parts of the soil (Zhang et al., 2021b). Forming fungal hyphae networks is conducive to the formation and stability of soil aggregates, which in turn increase the physical inaccessibility of soil organic carbon for microbial decomposition (Fabian et al., 2017). Nevertheless, we should be cautious of extending ecological network relationships for interpreting the variability and stability of the microbial community structure.

### Ecological Effects

Because the life cycle of these weeds is shorter compared with commonly grown perennial Ms., we hypothesized that there exists a unique microbial community in the soils of annual weeds. Based on our analysis, the soils devoted to weed growth and development have unique soil microbial properties as reflected by the microbial diversities and compositions (De Matos et al., 2019). The microbial properties significantly differed between the five weeds and Ms., which corresponded with differences elicited by inherent soil properties and the influence of plant traits on microbial communities (Shang et al., 2021). The significant increase in weed N and P indicates that they are highly competitive species in Ms. fields, which may be due to their strong environmental adaptability (Sardans et al., 2017). Moreover, they may release allelopathic substances, inducing changes in plant and soil microbial communities, thereby influencing soil nutrient levels and creating a positive ecological feedback loop (Wang et al., 2015). However, how these weeds regulate key rhizosphere effects on soil microorganisms remains unknown and should be the focus of future research.

## Conclusion

Changes exerted by five common weeds on the soil microbial community diversity, community composition, and microbial co-occurrence networks were investigated in an Ms. field in the NCP. Weeds with high stoichiometric flexibility, which have a high capacity for altering the elemental composition of their tissues/organs in response to environments with varied nutrient availability, generally have a greater potential to adapt or survive successfully. These weeds dramatically decreased bacterial and fungal diversities when compared with Ms. Shifts in the structural community compositions because the five weeds were tightly associated with changes in edaphic parameters (soil pH, NH_4_^+^-N, and NO_3_^−^-N) and plant traits (shoot and root biomass and R/S ratio). The abundances of pathogenic fungi were suppressed by the weeds, including *Nectriaceae* and *Pleosporaceae*, but were enriched in the rhizosphere soils of root-affected Ms. plants. Furthermore, weeds simplified the microbial co-occurrence network and possibly contributed to the higher assembly between microbial species. An integrated understanding of community assemblage will further clarify how weed species and soil microorganisms cooperate in weed–crop competition. Our work serves as an important resource for biological control in weed management practices as well as those of Ms. cultivation.

## Data Availability Statement

The datasets presented in this study can be found in online repositories. The names of the repository/repositories and accession number(s) can be found in the article/Supplementary Material.

## Author Contributions

CY, WT, and JS designed the study. JS, HG, SS, and FM participated in sample collection. CY performed the experiment and wrote the manuscript with the help of JQS, GY, YZ, and ZW. All authors contributed to the article and approved the submitted version.

## Funding

This study was funded by the China Agricultural Research System (No. CARS-34), the National Natural Science Foundation of China (32101434), the Natural Science Foundation of Shandong Province (ZR2020QC188), the Start Up Funds for High Level Talents of QAU (1120025), and the “First class grassland science discipline” program of Shandong Province, China.

## Conflict of Interest

The authors declare that the research was conducted in the absence of any commercial or financial relationships that could be construed as a potential conflict of interest.

## Publisher’s Note

All claims expressed in this article are solely those of the authors and do not necessarily represent those of their affiliated organizations, or those of the publisher, the editors and the reviewers. Any product that may be evaluated in this article, or claim that may be made by its manufacturer, is not guaranteed or endorsed by the publisher.
