# Supplementary material for: Weeds in the Alfalfa Field Decrease Rhizosphere Microbial Diversity and Association Networks in the North China Plain
Source: Front Microbiol. 2022 Mar 17;13:840774. doi: 10.3389/fmicb.2022.840774 (PMC8998637; doi:10.3389/fmicb.2022.840774)
Supplement: Supplementary file 1 [file Data_Sheet_1.DOCX]

**Table S1** The transitivity of co-occurrence networks of bacteria and fungi in root-associated soil of alfalfa and weeds.

| Transitivity | Bacteria | Fungi |
| --- | --- | --- |
| Ms | 0.75 | 0.46 |
| Ds | 0.56 | 0.53 |
| Ec | 0.45 | 0.39 |
| Aa | 0.3 | 0.35 |
| Po | 0.48 | 0.50 |
| Ca | 0.53 | 0.57 |


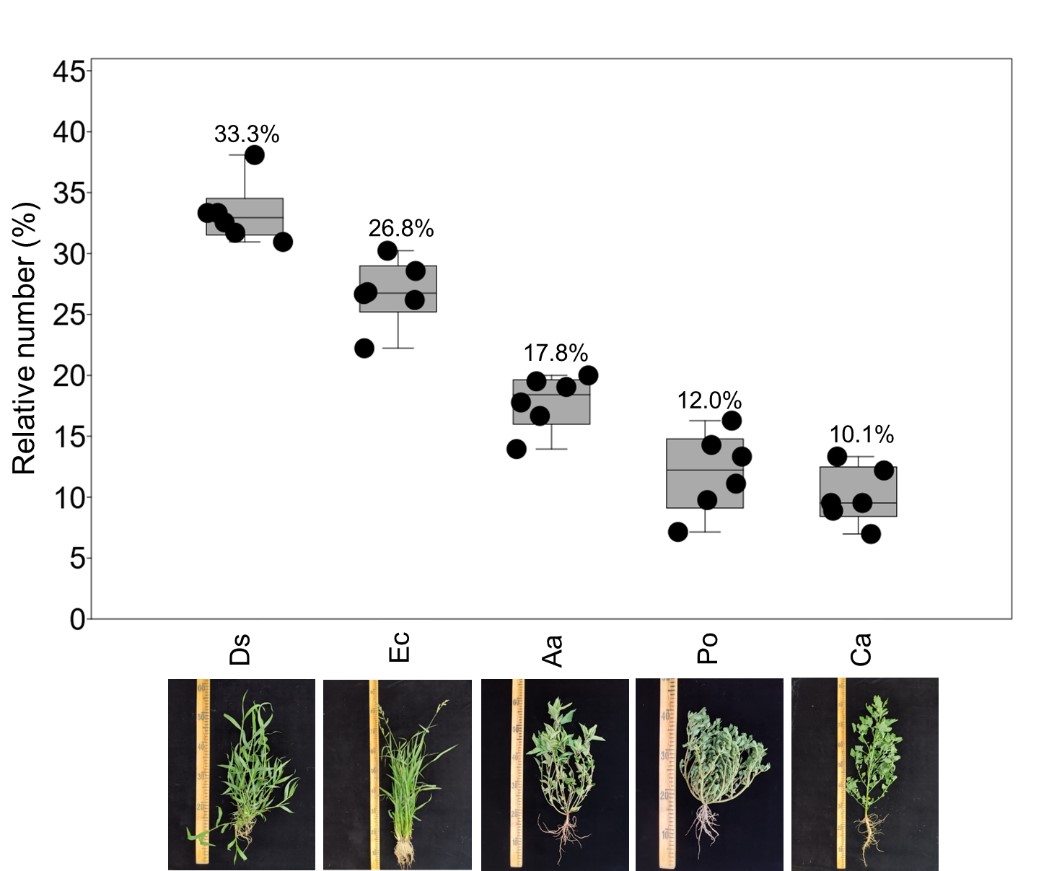


**Fig. S1.** The relative numbers of the five weed species in an alfalfa field.


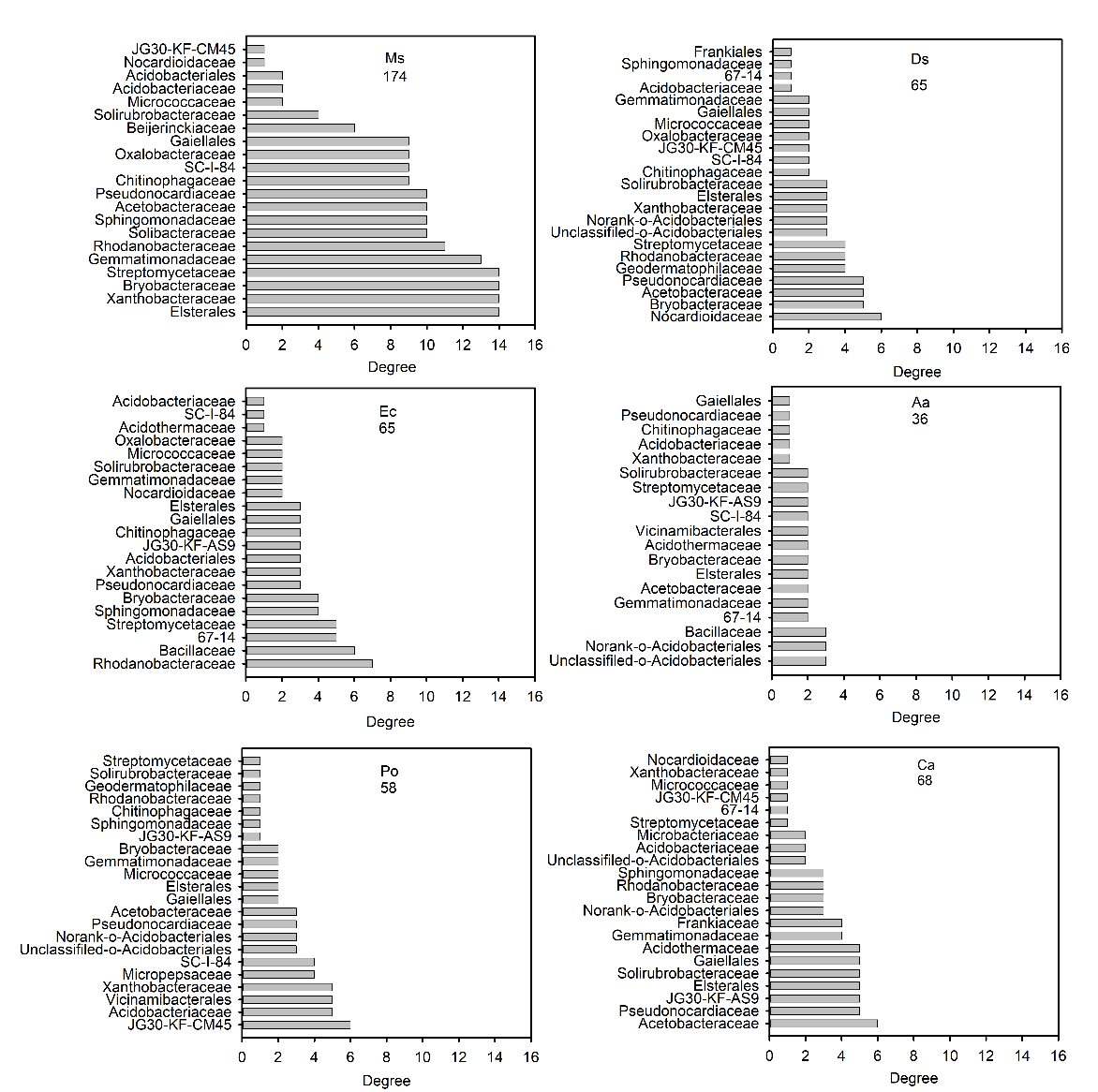


**Fig. S2.** The number of links for soil bacterial co-occurrence networks in root-associated soil of alfalfa and weeds.


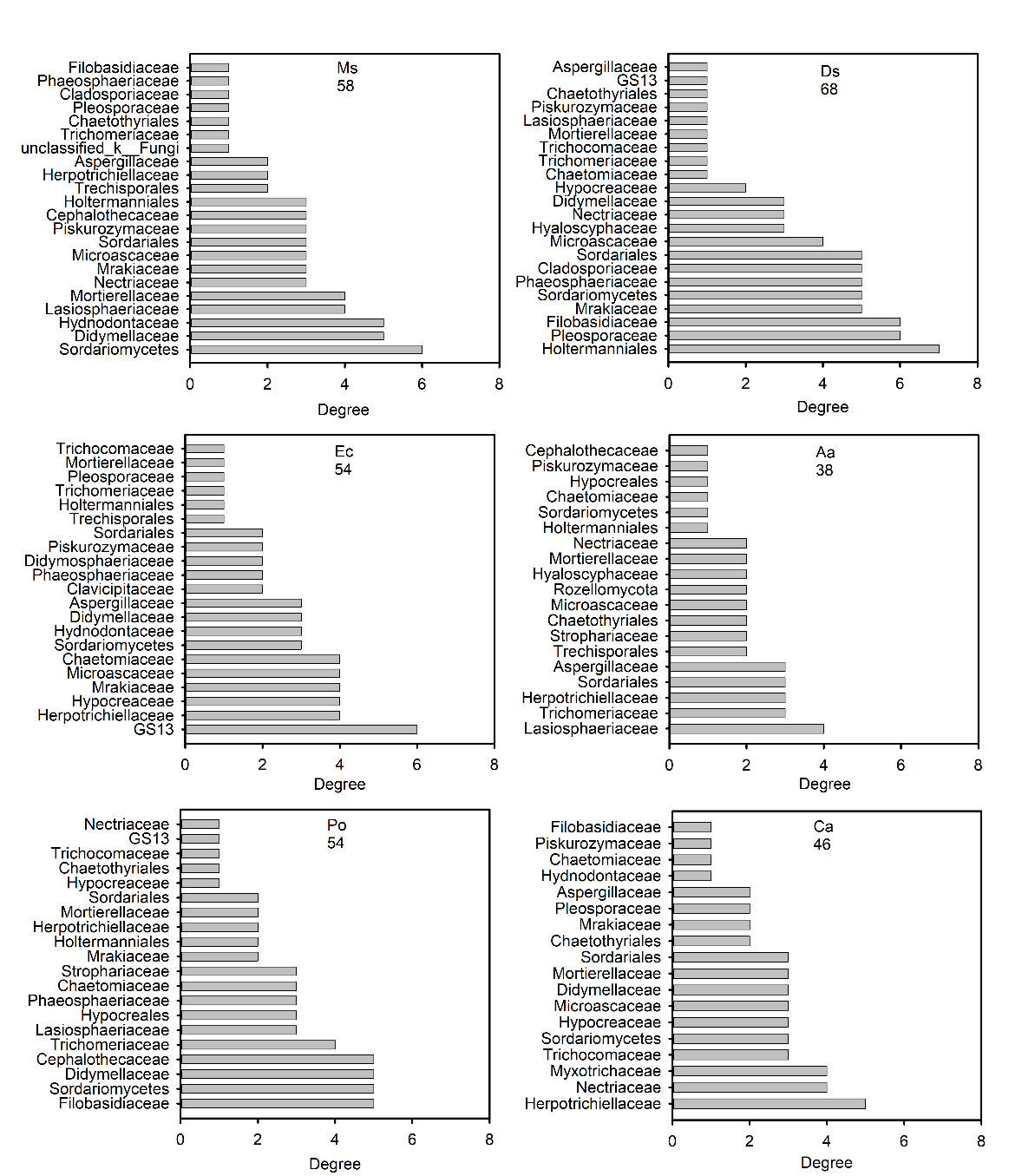


**Fig. S3.** The number of links for soil fungal co-occurrence networks in root-associated soil of alfalfa and weeds.
